# Reflectance Spectroscopy for Non-Destructive Measurement and Genetic Analysis of Amounts and Types of Epicuticular Waxes on Onion Leaves

**DOI:** 10.3390/molecules25153454

**Published:** 2020-07-29

**Authors:** Eduardo D. Munaiz, Philip A. Townsend, Michael J. Havey

**Affiliations:** 1 Department of Horticulture, University of Wisconsin, 1575 Linden Drive, Madison, WI 53706, USA; dominguezmun@wisc.edu; 2 Department of Forestry and Wildlife Ecology, University of Wisconsin, 1575 Linden Drive, Madison, WI 53706, USA; ptownsend@wisc.edu; 3 Agricultural Research Service of the United States Department of Agriculture (USDA) and Department of Horticulture, University of Wisconsin, 1575 Linden Drive, Madison, WI 53706, USA

**Keywords:** spectroscopy, onion, epicuticular wax, leaf biochemistry, PLSR, QTL, glossy, genetics, hentriacontanone-16, fatty alcohol

## Abstract

Epicuticular waxes on the surface of plant leaves are important for the tolerance to abiotic stresses and plant–parasite interactions. In the onion (*Allium cepa* L.), the variation for the amounts and types of epicuticular waxes is significantly associated with less feeding damage by the insect *Thrips tabaci* (thrips). Epicuticular wax profiles are measured using used gas chromatography mass spectrometry (GCMS), which is a labor intensive and relatively expensive approach. Biochemical spectroscopy is a non-destructive tool for measurement and analysis of physiological and chemical features of plants. This study used GCMS and full-range biochemical spectroscopy to characterize epicuticular waxes on seven onion accessions with visually glossy (low wax), semi-glossy (intermediate wax), or waxy (copious wax) foliage, as well as a segregating family from the cross of glossy and waxy onions. In agreement with previous studies, GCMS revealed that the three main waxes on the leaves of a wild type waxy onion were the ketone hentriacontanone-16 (H16) and fatty alcohols octacosanol-1 (Oct) and triacontanol-1 (Tri). The glossy cultivar “Odourless Greenleaf” had a unique phenotype with essentially no H16 and Tri and higher amounts of Oct and the fatty alcohol hexacosanol-1 (Hex). Hyperspectral reflectance profiles were measured on leaves of the onion accessions and segregating family, and partial least-squares regression (PLSR) was utilized to generate a spectral coefficient for every wavelength and prediction models for the amounts of the three major wax components. PLSR predictions were robust with independent validation coefficients of determination at 0.72, 0.70, and 0.42 for H16, Oct, and Tri, respectively. The predicted amounts of H16, Oct, and Tri are the result of an additive effect of multiple spectral features of different intensities. The variation of reflectance for H16, Oct, and Tri revealed unique spectral features at 2259 nm, 645 nm, and 730 nm, respectively. Reflectance spectroscopy successfully revealed a major quantitative trait locus (QTL) for amounts of H16, Oct, and Tri in the segregating family, agreeing with previous genetic studies. This study demonstrates that hyperspectral signatures can be used for non-destructive measurement of major waxes on onion leaves as a basis for rapid plant assessment in support of developing thrips-resistant onions.

## 1. Introduction

Foliar spectroscopy uses reflectance or absorption features associated with molecular bonds to both rapidly and non-destructively estimate amounts of specific chemicals in living plant tissue [1,2,3,4]. Reflectance spectroscopy, also known as hyperspectral sensing, consists of continuous measurements across a broad spectral range, e.g., every 3 nm in the UV/visible to near-infrared (VNIR, 350–1000 nm) or to shortwave infrared (VSWIR, 350–2500 nm). Spectroscopy has been successfully used to estimate physiological and biochemical features of plants. Chlorophyll [3,5,6] and carotenoids [7,8] have received the greatest attention because of their importance in plant metabolism and distinctive absorption features. Spectroscopy on green leaves has also been used to measure nitrogen concentration in tissues from a range of crops [9,10,11], as well as physiological parameters such as rates of ribulose-1,5-bisphosphate-carboxylation as an estimate of photosynthetic capacity [12] and other constituents such as phenolics that are important for plant defense [1,13,14,15]. Hyperspectral data have been utilized to differentiate the reflectance fingerprints of susceptible versus resistant phenotypes for the pathogen *Cercospora beticola* in sugar beet [16], damage by *Thrips tabaci* L. (thrips) on cabbage [17,18,19], and numbers of microsclerotia of the fungus *Macrophomina phaseolina* (Tassi) Goid on soybean [18], supporting spectroscopy as a selection tool for plant breeding.

To our knowledge, hyperspectral data have not been used to estimate epicuticular wax components important for insect interactions, although it is well documented that epicuticular waxes influence reflectance spectra [20]. Epicuticular waxes are hydrophobic organic molecules that accumulate on the surface of plant leaves and stems. Natural variation exists for wax components among plants [21,22]. In *Arabidopsis thaliana* L., wax composition is predominantly comprised of alkanes, ketones, and secondary alcohols [23]. In leek (*Allium ampeloprasum* L.), leaf waxes are fatty acids, ketones, alkanes, and aldehydes with the ketone hentriacontanone-16 (H16) as the most prevalent wax [24]. In onion (*Allium cepa* L.), the main epicuticular waxes are a ketone, fatty alcohols, and alkanes [25,26]. Onion plants vary for amounts of epicuticular waxes and are visually classified as glossy (relatively low wax), semi-glossy (intermediate amount of wax), or wild-type waxy (copious wax) [25,26,27,28]. Glossy and semi-glossy onions experience significantly less feeding damage from thrips relative to waxy onions [25,26,29,30,31]. Previous studies used GCMS and revealed that the amounts of the three main waxes on onion leaves (the ketone hentriacontanone-16 (H16) and fatty alcohols octacosanol-1 (Oct) and triacontanol-1 (Tri)) are significantly higher on the foliage of waxy than glossy or semi-glossy phenotypes. Munaiz et al. [26] showed that semi-glossy onions can possess as much total wax as waxy phenotypes due to relative proportions of individual waxes, and these plants suffer less feeding damage from thrips.

GCMS is a relatively labor intensive and expensive approach to measure leaf waxes. VSWIR spectroscopy is a powerful non-destructive approach that may be useful for leaf wax measurements. To our knowledge reflectance spectroscopy has not been used for genetic analysis in agricultural crops, neither for characterization of very long chain derived fatty alcohols as epicuticular waxes. Spectroscopy was used for quantitative analysis in the forest tree *Populus* [32] to identify candidate locus controlling the production of salicinoid phenolic glycosides. Spectroscopy was also used in poplar for secondary metabolites identification [33]. In this study, we measured amounts and types of epicuticular waxes on leaves of phenotypically diverse onions using GCMS and spectroscopy to determine the spectral characteristics of leaf waxes and assess the efficacy of spectroscopy as a breeding tool to develop thrips-resistant onions.

## 2. Results and Discussion

Accessions were selected based on visually glossy, semi glossy, or waxy phenotypes and showed significant differences for amounts of individual waxes (Table 1, Table 2 and Table 3). These accessions also provided optimal spectroscopic phenotypic ranges for H16, Oct, and Tri between 0–2.2, 0–0.77, and 0–0.41, respectively (Table 4).

### 2.1. Phenotypic Variation for the Three Major Epicuticular Waxes

H16 is the most abundant epicuticular wax on the leaves of wild type waxy onions [25,26] and waxy DH2107 had the largest mean adjusted peak area of 2.015 (Table 2). Adjusted mean peak area for semi-glossy plants from PI 264320 was 1.091, followed by the other semi-glossy accessions ranging from 0.533 to 0.648 (Table 2). Glossy B9885 had a slightly higher adjusted peak area for H16 at 0.700, and glossy ‘Odourless Greenleaf’ (OGL; PI 289689)) was unique with essentially no H16 at 0.002 (Table 2). The segregating F2 family had adjusted peak areas for H16 ranging from 0.00 to 2.67 with a mean of 0.84 (Supplementary Figure S1).

Glossy OGL and semi-glossy 264,320 had the largest amounts of Oct with a mean adjusted peak areas of 0.546 and 0.586, followed by the semi glossy and glossy accessions (Table 2). Waxy DH2107 had a relatively low amount of Oct at 0.496, in agreement with Damon et al. [25] and Munaiz et al. [26]. Amounts of Oct were not consistent with visual phenotypes, for example glossy OGL and waxy DH2107 had statistically the same amount of Oct. The phenotypic distribution in the segregating F2 family for Oct ranged from 0.00 to 0.39 adjusted peak areas with a mean of 0.14 (Supplementary Figure S1).

OGL foliage accumulated no Tri. Semi-glossy B5351 (0.075) and glossy B9885 (0.140) had lower amounts of Tri compared with the semi glossy accessions PIs 264320, 546192, and 546115, which ranged from 0.195 to 0.247 mean adjusted peak areas (Table 2). Waxy DH2107 had a slightly larger mean adjusted peak area of Tri at 0.260. The phenotypic distribution for Tri in the segregating F2 family ranged from 0.00 to 0.25 with a mean at 0.08 (Supplementary Figure S1).

### 2.2. Proportions of Amounts of Individual Waxes to Total Wax

Relative proportion of H16 to other wax components is associated with visual leaf phenotypes [24,25,26,36] and the onion accessions differed significantly for relative proportions of specific waxes [26]. Waxy DH2107 possessed the largest proportion of H16 at 65.0% (Table 3) and lower proportions of fatty alcohols Oct at 16.0% and Tri at 8.4%. The semi-glossy PIs had relatively similar proportions of H16 ranging from 36.8% for PI 546115 to 52.9% for B5351. Glossy OGL accumulated essentially no H16 and higher proportions of the fatty alcohols Oct at 70.7% and Hex at 19.2%, and a relatively high proportion of the alkane 1-ethenyloxy octadecane at 9.6%. Tri ranged from 0.00% (OGL) to 15.9% among all accessions with PI 546115 having the highest proportion of this wax. OGL has a unique wax profile compared to all other waxy, semiglossy, and the other mutant glossy B9885 [34].

### 2.3. Variation of Reflectance for Purified Chemical Standards

**Figure 1:**
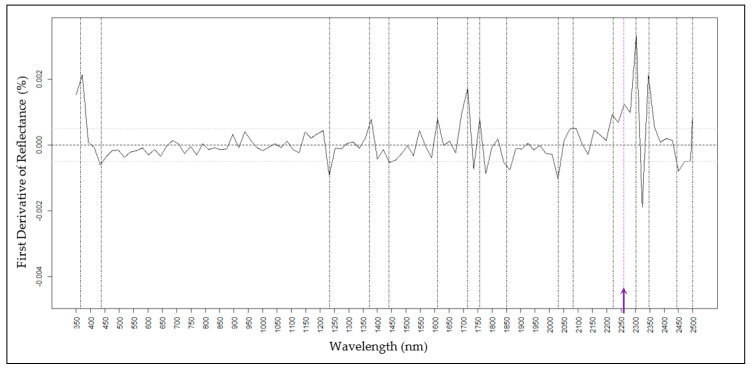
First derivative reflectance of pure Hentriacontanone-16. Black vertical lines indicate the absorption features associated with H16. Purple dash line with an arrow on the base indicates a unique absorption feature for H16.

**Figure 2:**
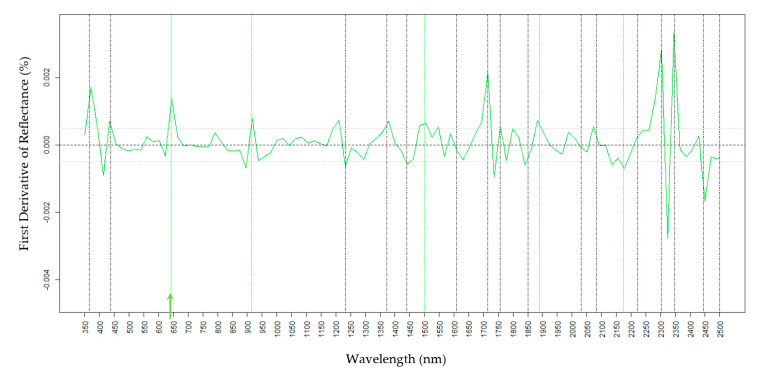
First derivative reflectance of pure Octacosanol-1. Black vertical lines indicate absorption features associated with H16, Octacosanol-1, and Triacontanol-1. Green lines are Octacosanol-1 specific. The green arrow at 645 nm depicts a unique absorption feature for Octacosanol-1.

**Figure 3:**
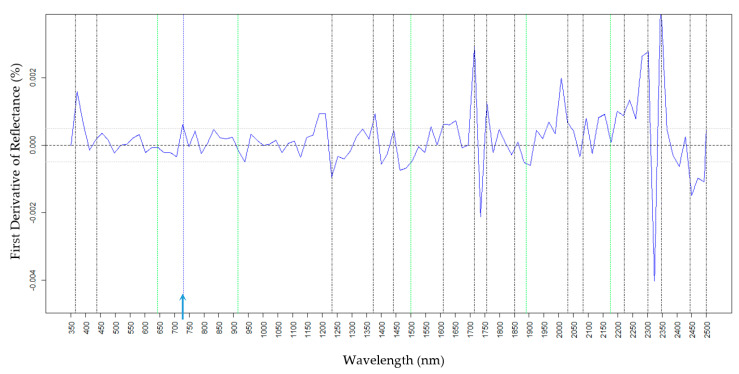
First derivative reflectance of the pure Triacontanol-1. Black vertical lines indicate absorption features common with H16 and Octacosanol-1. Green lines indicate absorption features unique to Octacosanol-1 and blue lines depicted with a blue arrow on the base are Triacontanol-1 specific.

Chemical standards were used to determine the reflectance spectroscopic absorptions for H16, Oct, and Tri, the three most abundant epicuticular waxes on the foliage of wild-type waxy onion. For H16, reflectance peaks occurred at wavelengths of 365, 437, 1233, 1372, 1440, 1610, 1714, 1757, 1850, 2031, 2082, 2222, 2302, 2348, 2445, and 2499 nm (Figure 1) with a unique wavelength at 2259 (Figure 1, purple line). Unique spectral signatures distinguish the presence versus absence of a specific wax and can be considered as a single spectral variation (SSV). For Oct, a unique spectral feature was detected at 650 nm and spectral features at 915, 1500, 1890, and 2175 nm were shared with Tri (Figure 2 and Figure 3). For Tri, a unique absorption feature was revealed at 730 nm (Figure 3). Tri did not have a major reflectance peak at 437 nm compared to H16 and Oct, and wavelengths within the range 2020–2030 nm overlapped between Tri and H16, but not with Oct (Figure 3). These results indicate that there are unique spectral signatures distinguishing H16, Oct, and Tri. Of note, there are strong spectral signals for all waxes at 1714–1757 nm and 2302–2348 nm.

### 2.4. Predictions of Wax Components Using PLSR Models

For the calibration of spectral data, we evaluated waxy DH2107, glossy OGL with essentially no H16 and Tri, and semi-glossy PI 546303 with a lower amount of H16, PI 546115 with a higher amount of Tri, PI 546192 with a higher amount of Oct, and PI 264320 with a lower amount of H16, all relative to waxy DH2107. Quality control of each spectral measurement was visually assessed, and those with abnormalities due to a technical error or low reflectance were removed and therefore not included in the downstream prediction analysis [37,38].

#### 2.4.1. H16

**Figure 4:**
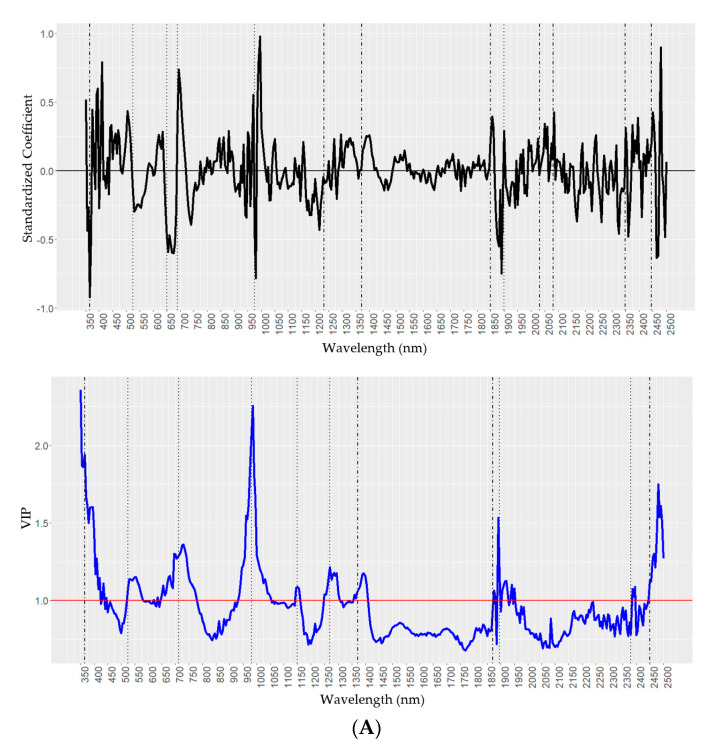

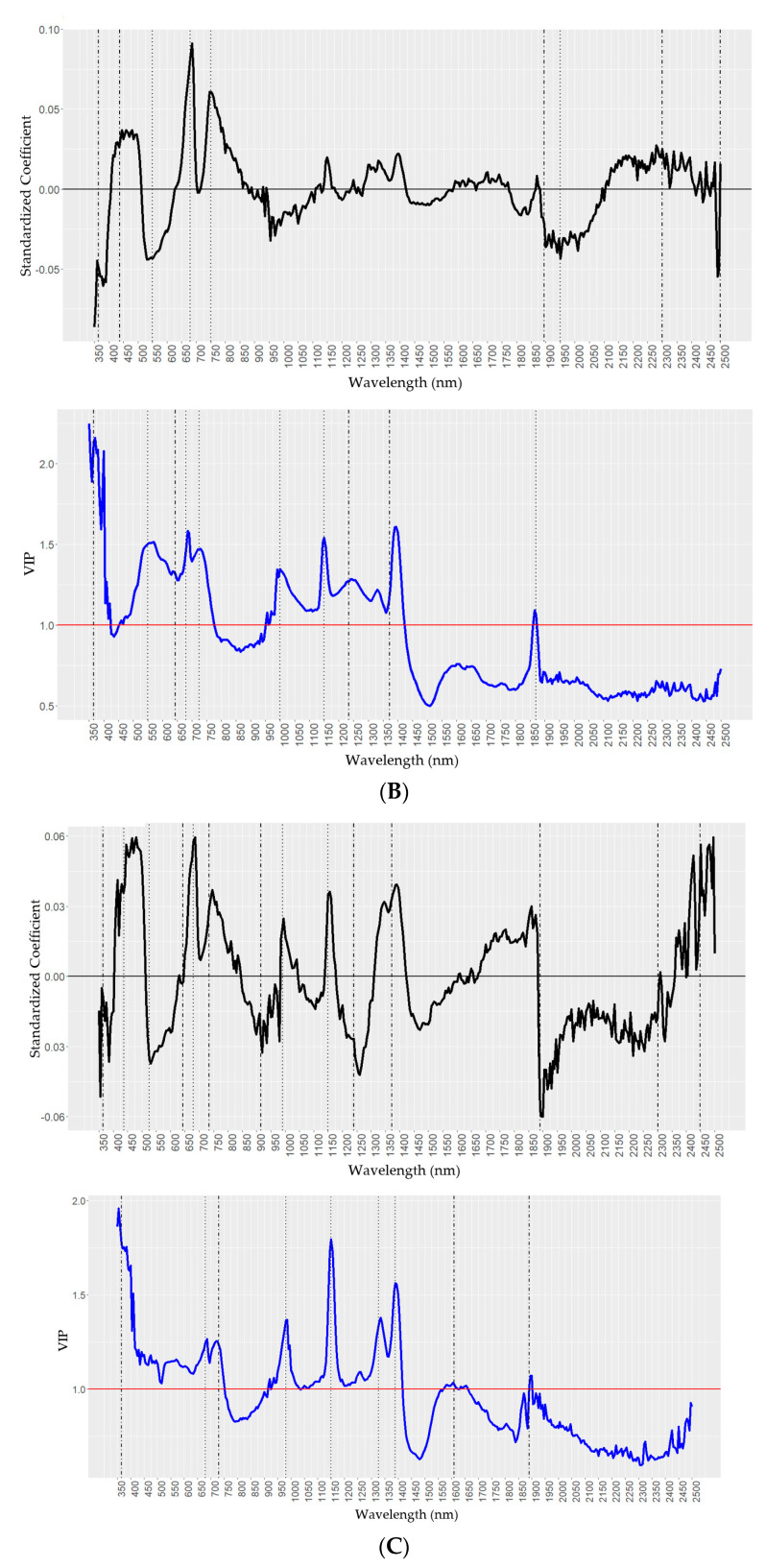
Qualitative parameters variable importance of projection (VIP, blue line) and standardized coefficients (black line) for fatty alcohols. (**A**) Hentriacontanone-16 (H16), (**B**) Octacosanol-1 (Oct), and (**C**) Triacontanol-1 (Tri) measured on onion leaves. VIP peaks above the red line indicate the threshold more relevant in the partial least-squares regression (PLSR) model and wavelengths (nm) for the detection of each wax component. In each panel vertical dotted lines indicate absorption features detected only in living tissue, and vertical dot dash line are absorption features detected both in living tissue and the chemical standards for H16, Oct, and Tri as shown in Figure 1, Figure 2 and Figure 3, respectively.

Predictions for the amounts of H16 on the foliage of the seven onion accessions using partial least-squares regression (PLSR) models showed the highest coefficient of determination (R2) at 0.86 (Table 4), root-mean-square error (RSME) of 0.182 and a relative RMSE at 8.2% (of a data range of 0.00–2.20 of H16). The prediction accuracy of the PLSR model on external data that included a segregating family yielded an R2 of 0.72, RMSE was slightly higher at 0.304, and relative RMSE at 12.4% (range 0.00–2.41), indicating that the prediction model for H16 was robust. The scatter plot of the best regression showed significant correlations (Supplemental Figure S2). The randomly dispersed residuals along the horizontal axis indicated that the PLSR model is well suited to predict H16 values (Supplemental Figure S2). Higher residual values suggest that the model may overestimate higher values of H16 amounts. Density distribution of the F2 segregating family for the two data sets showed a similar mean value at 0.84 and 0.83 for the GCMS and spectroscopy prediction, respectively (Supplementary Figure S1). Variable importance in projection (VIP) scores estimate the relevance of each wavelength used in the PLS regression model. A VIP greater than 1 indicates the most important wavelength in the model (Figure 4, A-bottom, blue line). VIP revealed that the most relevant wavelengths measured in living tissue were 365 nm, 525 nm, 712 nm, 980 nm, 1150 nm, 1270 nm, 1372 nm, 1860 nm, 1895 nm, 2380 nm, and 2450 nm (Table 5). Interestingly, wavelengths 365 nm, 1372 nm, 1860 nm, and 2450 nm (Figure 4A) were aligned with peaks identified in the H16 pure standard measurements (Figure 1) suggesting that these wavelengths were more relevant for highest prediction accuracy for H16. Standardized coefficients with higher absolute values for given wavelengths have a greater influence on H16 prediction (Figure 4, A-top, black line). Standardized coefficients detected most relevant wavelengths measured in living tissue at 525 nm, 650 nm, 690 nm, 975 nm, and 1900 nm. In addition, wavelengths 365 nm, 1233 nm, 1372 nm, 1850 nm, 2031 nm, 2082 nm, 2348 nm, and 2445 nm (Table 5, Figure 4A) were also detected and aligned with those revealed by the H16 pure standard measurements (Figure 1). These quality parameters revealed that the most relevant wavelengths for highest prediction accuracy for H16. Curran [4] reported that absorption features at 970 nm, 1200 nm, 1400 nm, and 1940 nm were the result of the bending and stretching of the O-H bond in water and other molecules. For H16, an important absorption feature overlapped with water absorption occurring in the 900–1000 nm spectral range [4,39]. The unique 2259 nm feature identified for the purified standard was not an important contributor to the chemometric model due to the relative diagnostic strength of other spectral features also identified in the standard (Figure 4A).

#### 2.4.2. Oct

For Oct, cross validation of the PLSR model revealed a relatively high R2 at 0.67, root-mean-square error of the model at 0.108, and the relative RMSE at 14.0% (Oct data range 0.00–0.77). Prediction performance the external validation data that included the segregation family improved slightly with a coefficient of determination at 0.70 (RMSE 0.102) and similar relative RMSE of 15.5% (range of 0.00–0.67), indicating the robustness of the prediction model. For Oct, the density distribution of the F2 progeny from the two data sets showed a similar mean value at 0.143 and 0.145 for the GCMS and spectroscopy prediction, respectively, and a median slightly higher for the spectroscopy prediction (Supplementary Figure S1, Yellow dashed-line). Important spectral features for Oct overlapped with H16 close to wavelengths of 365 nm, 437 nm, 1233 nm, 1372 nm, 1440 nm, 1610 nm, 1714 nm, 1757 nm, 1850 nm, 2031 nm, 2082 nm, 2222 nm, 2302 nm, 2348 nm, 2445 nm, and 2499 nm (Figure 1) suggesting that there is an additive effect of multiple spectral features for wax components. However, the intensity of reflectance among these signature wavelengths varies among wax components (Figure 1, Figure 2 and Figure 3). A unique absorption feature for Oct was revealed at wavelength 645 nm, and common wavelengths with Tri at 915 nm, 1500 nm, 1890 nm, and 2175 nm. VIP scores indicated that most relevant wavelengths measured in living tissue were 365 nm, 550 nm, 645 nm, 680 nm, 725 nm, 1000 nm, 1150 nm, 1233 nm, 1372 nm, and 1870 nm (Table 5). Interestingly, wavelengths 365 nm, 645 nm, 1233 nm, and 1372 nm (Figure 4B) were aligned with peaks identified with the Oct pure standard measurements (Figure 2) suggesting that these wavelengths were more relevant for the highest prediction accuracy for Oct. The standardized coefficient revealed wavelengths at 365 nm, 437 nm, 550 nm, 680 nm, 750 nm, 1890 nm, 1950 nm, 2300 nm, and 2499 nm associated with the amounts of Oct. Of these wavelengths, 365 nm, 437 nm, 1890 nm, 2300 nm, and 2499 nm were in synteny with the ones revealed with Oct pure standards measurements indicating that of highest importance for Oct is the prediction accuracy. Importantly, the Oct unique absorption 645 nm was detected in living tissue and with the Oct pure standard, confirming its importance for Oct chemical detection. In addition, bands at 913 nm and 1890 nm were also relevant for the prediction models given the large absolute values of the coefficient at these wavelengths.

#### 2.4.3. Tri

For Tri, the PLSR cross-validation model had an R2 of 0.48, root-mean-square error of the model was 0.068, and the relative RMSE was 16.4% (Tri data range of 0.00–0.41). Prediction performance on the external data set validation showed a similar R2 at 0.41 (RMSE 0.072) and the relative RMSE was the highest for the three wax PLSR models at 20.7% (range 0.00–0.35; Table 4). Since the R2 obtained within the cross-validation dataset was almost the same to the external validation dataset this is an indication of the robustness of the regression model. The lower concentrations (and thus more compressed data range) of Tri relative to H16 and Oct may lead to a lower R2 for prediction models. The density distributions for Tri and the segregating F2 family showed a good overlap between the predictions from GCMS and spectroscopy measurements with a mean at 0.88 and 0.85 (Supplementary Figure S1) and an overlap of the medians (Supplementary Figure S1, dashed lines), indicating the robustness of the method. A unique absorption feature for Tri was detected at wavelength 730 nm as indicated by the vertical blue dotted line in Figure 3. For Oct and Tri, wavelengths of 915 nm, 1500 nm, 1890 nm, and 2175 nm were in common (Figure 2 and Figure 3, vertical green lines). For Tri, VIP scores (Figure 4C, blue line) revealed a wavelength 365 nm, 680 nm, 730 nm, 980 nm, 1150 nm, 1328 nm, 1390 nm, 1610 nm, and 1890 nm (Table 5). In addition, wavelengths 365 nm, 730 nm, 1610 nm, and 1890 nm (Figure 4C, bottom) aligned with those revealed with the Tri pure standard measurements (Figure 3). The standardized coefficients (Figure 4C, top) revealed the importance of 525 nm, 680 nm, 991 nm, and 1150 nm for this wax. In addition, wavelengths 365 nm, 437 nm, 643 nm, 730 nm, 915 nm, 1233 nm, 1372 nm, 1890 nm, 2302 nm, and 2449 nm were also detected, which aligned with the Tri pure standard (Figure 3) suggesting that these are more relevant for this wax highest prediction accuracy. The unique Tri fingerprint, 730 nm, stand out as an important spectral feature for this wax, also present as a high absolute value of the VIP and chemical standard (Figure 4C, top, black line). Interestingly, 915 nm and 1890 nm spectral wavelengths are singularly important for the two fatty alcohols Tri and Oct prediction models. These results revealed important absorption features across the visible-near-infrared-SWIR spectra for estimation of the amounts of the three main epicuticular waxes on onion foliage.

### 2.5. Genetic Mapping Using Spectrometric Measurements of H16, Oct, and Tri

A genetic map was previously developed using an F2 family from the cross of glossy B9885 by waxy B8667 and the glossy phenotype was associated with a recessive locus (*gl*^wp^) on chromosome 8 [34]. Glossy foliage in *A. fistulosum* is also conditioned by a single recessive locus [40]. This same B9885 by B8667 segregating family was used for the genetic analysis of spectroscopic measurements. Quantitative analysis of H16 amounts using the predicted values revealed a major quantitative trait locus (QTL) on chromosome 8 at 39.0 cM with a logarithm of odds (LOD) score of 4.9 and *p* < 0.001. The closest single nucleotide polymorphism (SNP) was isotig19082_1721 at 41 cM, which explained 21% of the phenotypic variation (Table 6), and mapped to the same genomic region as the glossy *gl*^wp^ locus [34]. This QTL had an additive effect of 0.10 of the allele from waxy parent B8667 and a dominance effect 0.14 to increase the H16 amounts. Analysis of the fatty alcohol Oct predicted with spectroscopy revealed a QTL on chromosome 8 at 41.1 cM that explained 21% of the phenotypic variation at LOD 3.8. This QTL has an additive allelic substitution effect of 0.36 from the waxy parent and a dominant effect 0.31 increasing amounts of Oct. We also detected the same QTL for the fatty alcohol Tri measured with spectrometry (Table 6) with the closest SNP being isotig19082_1721. The phenotypic variation explained was 36.4% at a LOD score of 9.1. QTLs affecting the amounts of Oct and Tri detected by spectrometry explained a larger proportion of the phenotypic variation than GCMS (Table 6). In addition, the LOD score for Tri was higher with spectroscopy than GCMS at 9.1 versus 5.5, respectively, and the prediction model using hyperspectral tools with the R2 cross-validation dataset was almost identical to the external validation dataset. These results demonstrate that mapping and genetic effects using predicted spectroscopic values (Table 6) detected the same major QTL as GCMS [34].

## 3. Materials and Methods

### 3.1. Plant Materials

Seven onion accessions were used to measure the amounts and types of epicuticular waxes on leaves by GCMS and spectroscopy: plants from the cultivar ‘Odourless Greenleaf’ (OGL; USDA plant introduction (PI) 289689) and inbred B9885 (PI 546303) have visually glossy foliage; PIs 264320, 546115, and 546192 and inbred B5351 (selected from the cultivar ‘Colorado #6′) have visually semi-glossy foliage; and doubled haploid (DH) 2107 [35] has visually waxy leaves [25]. Seeds were sown into a soilless mix (Metro-Mix, Sun-Gro Horticulture, Agawam, MA, USA) in 96-well trays in a greenhouse with supplemental lighting at 14 h days and constant temperature of 24 °C. After 20 days, plants transferred to 35.6 cm diameter pots and arranged in a greenhouse in a completely randomized design with three replications. Plants were watered using an automatic flooding system activated once per day that supplied 1/4 Hoagland’s solution [41]. Five consecutively aged leaves on each plant were used for spectral measurements and for the determination of wax composition by GCMS. This experiment was repeated twice.

A segregating F2 family from the cross of glossy B9885 with waxy B8667 [34] was grown in the field in 2017 at the Dean Kincaid Farm (Palmyra, WI, USA) under normal production conditions and bulbs were harvested and stored at 4–7 °C in the dark for 4 months. One hundred F2 bulbs were planted into Metro-Mix in 35.6-cm diameter pots and grown in a greenhouse at the UW Arlington Research Farm under 12-h days and temperatures of 27 °C and 22 °C nights. Seven weeks after bulb planting, the middle longitudinal section of the third leaf on each of the 94 plants was used for spectral measurement. Since spectral readings are non-destructive, two consecutive leaf segments (2.5 cm approximately) of the same leaf were sampled for GCMS.

### 3.2. Spectral Measurements of Onion Leaves

Spectral reflectance of onion leaves was measured using a PSR+ 3500 high resolution full range portable spectroradiometer (Spectral Evolution, Lawrence, MA, USA). Each measurement utilized a bifurcated fiber optic, in which one fiber illuminated the foliage with light from a tungsten-halogen source and the other transmitted the reflected light to the detector. The fiber optic cable was pointed perpendicularly to the onion leaf and wrapped with a black cloth (5 cm × 5 cm) to avoid light contamination. Relative reflectance was calculated using a 99% spectralon panel (Labsphere, North Sutton, NH, USA). Reflectance measurements were taken on four leaves on each of the three plants per accession and were recorded at four consecutive points along the middle longitudinal region of each leaf. The four reflectance measurements per leaf were then averaged. For spectroscopic measurements of the segregating F2 progenies, four reflectance measurements were taken on the third leaf of each plant as described above, and these measurements were averaged to obtain one spectral reading per F2 plant.

### 3.3. Spectral Measurements of Chemical Standards

Pure standards of the three main epicuticular waxes on onion foliage (H16 (>95%), Oct (99%), and Tri (>99%)) were used for spectral calibration (Tokyo Chemical, Portland, OR, USA). Each standard powder was poured on a device with a conical frustum well (6.3 mm × 12.6 mm × 10.0 mm) mounted with a lens 12.7 mm diameter and 3 mm thick (Thorlabs, Newton, NJ, USA), and lightly shaken to distribute the sample. Measurements were conducted with the fiber optic cable held perpendicularly to each sample and relative reflectance was calculated using the 99% spectralon panel. Reflectance was recorded four times at four consecutive points so that each spectrum was the average of the four reflectance measurements.

### 3.4. Gas Chromatography Gas Spectroscopy (GCMS)

Samples for GCMS of epicuticular waxes were harvested as described above, weighed, and docosane (Sigma-Aldrich, St. Louis, MO, USA) dissolved in high-performance liquid chromatography (HPLC) grade chloroform (Fisher Scientific, Hampton, NH, USA) was added onto the surface of each leaf sample at 100 µg per g of leaf fresh weight. Leaf pieces were then submerged into HPLC-grade chloroform (Sigma-Aldrich) for 1 min and discarded. Chloroform was evaporated under a fume hood and the dried residue was dissolved in 500 μL chloroform, 600 μL acetronitrile (Fisher Scientific, HPLC grade), 210 μL N,O bis(trimethylsilyl)trifluoroacetamide BSTFA (Sigma-Aldrich, 1% trimethyl-chlorosilane, HPLC/GC grade), and treated at 80 °C for 30 min for derivatization. Waxes were identified and quantified using GCMS instrument QP2010 (Shimadzu, Columbia, MD, USA) with a capillary GC column (SH-Rxi-5Sil MS; 30 m long; 0.30 mm i.d.; df = 0.25 μm), and on-column injection at 250 °C, column oven temperature 150 °C constant for 10 min, ramp 10 °C per min. to 300 °C, constant for 10 min. Helium was the carrier at a flow rate of 1.0 mL per min with primary pressure of 700 kPa. Tandem MS was equipped with a detector (GCMS-QP2010) with ion source range, 35–600 *m/z*, for identification of the wax components. The detection MS interface and ion source temperatures were 290 and 260 °C respectively, and a split ratio of 20. The amounts of individual waxes are reported as mean peak areas adjusted to the internal docosane standard, and therefore represent peak areas of each wax per gram of leaf fresh weight.

### 3.5. Model Development

Data were partitioned randomly with two-thirds for calibration and one-third for validation, with the constraint of drawing evenly from all quartiles. Validation data were never used for model building. The same calibration and validation data sets were used for all chemicals. Spectral data in the native format were provided at 1 nm intervals, interpolated from the native 1.5–3.8 nm resolution; however, we used data at 5 nm intervals (350 nm, 355 nm, 360 nm, etc., to 2500 nm) as preliminary testing showed no benefit of using every wavelength. Partial least-squares regression (PLSR) [33,42,43,44,45] was used to estimate the amounts of H16, Oct, and Tri as a function of 5 nm interval spectra. PLSR is widely used in chemometrics (chemical discipline lead by data driven deployment of chemical analysis by applying mathematical and statistics methods), and operates by iteratively transforming predictor and response variables to identify latent vectors that maximize the covariance between independent (wavelengths) and dependent (biochemical analytic) variables while simultaneously maintaining the constraint of being orthogonal to the previously determined factors. PLSR generates a coefficient of multiple determination for every wavelength, which when applied to a new dataset generates a prediction for the measurement. We utilized the predicted residual sum of squares (PRESS) [46] over 500 permutations of the 66% calibration data split 90/10 to select the number of latent vectors that minimized PRESS. The cross-validation R2 was the result of 500 permutations. Once a model was determined, validation R2 was determined from the application of the coefficients of determination to the independent validation data set (33%) that included the segregating F2 family.

### 3.6. Data Analysis

Statistical analyses were performed according to the experimental design in R studio [47]. Accessions and replicate were considered as fixed effects. Analysis of variance was used for fixed effects with the anova function and Lmer package with function lm for model analysis. Significant differences for wax composition among accessions were tested using the package emmeans and Tukey’s honest significant difference at *p* = 0.05. The model used to estimate accession means was y_ijk_ = µ + g_i_ + t_j_ + gt_ij_ + ε_ijk_; where µ is the overall mean, gi is the fixed effect of the ith onion accession, tj is the fixed effect of the jth repetition, gtij is the effect of the ijth accession by repetition, and εijk are residuals.

In addition to R2 and root-mean-square error (RMSE) estimates using the package hydroGOF and rmse function, the model coefficients provided information on areas of the visible-through-shortwave-infrared (VSWIR) spectrum that are most predictive of the independent variable of interest [47]. We displayed the standardized PLSR coefficients (centered and standardized) enabling comparison across wavelengths because of differences in magnitude in different areas of the spectrum, e.g., low reflectance in blue and red, high reflectance in near infrared). We also used the VIP (variable importance of projection) statistic as described in Wold [48], which evaluates the importance of individual wavelengths in explaining the variation in both the response and predictor variables based on partial-R2; larger VIP scores indicate greater contribution by specific wavelengths to the predictive model. The important wavelengths (either using standardized coefficients or VIP) indicate potential molecular absorption features that enable detection of the chemical compound. We interpreted these based on the literature, as well as first-derivative spectra of standards of each chemical.

### 3.7. QTL Analysis

Quantitative analysis of spectroscopic predicted values for H16, Oct, and Tri used the genetic map previously reported by Munaiz and Havey [34]. Interval mapping with pseudo markers imputed at 1 centimorgan (cM) was used the R/qtl package [49]. To account for neighboring markers Haley Knott regression was used with parameters 10 cM window, and ten markers as covariates [50]. Significant association used the 95% significance LOD threshold after 1000 permutations. Stepwiseqtl function was implemented at 0.05 significance and effects of candidate QTLs were estimated using makeqtl and fitqtl in R. The fitted model provided the percentage of the variation explained by the QTLs and estimated allelic substitution effects.

## 4. Conclusions

Reflectance spectroscopy was used for non-destructive measurement of amounts of major epicuticular waxes on onion leaves and revealed distinct absorption features in the visible (645 nm), near infrared (730 nm), and SWIR (2259 nm) for Oct, Tri, and H16, respectively. We observed high coefficients of validation (0.84, 0.67, and 0.48) and good quality overlap between GCMS and spectroscopic measurements. We also demonstrated that spectroscopy can be used reliably for identification of unique wax phenotypes such as OGL and for genetic analyses of wax amounts confirming a QTL on chromosome 8 associated with amounts of Oct, Tri, and H16. Hyperspectral analyses should be an effective tool for non-destructive measurements of amounts of specific epicuticular waxes on onion foliage towards the development of thrips resistant onions. Since we were able to identify unique features for each wax, spectroscopy can be used for selection by identifying presence–absence variation of SSV, for instance selecting lines in the field with no H16 or Tri. Another strategy to develop thrips resistance in onions could be recurrent selection for lower levels of H16 and higher amounts of fatty alcohols using spectroscopy as a non-destructive, in situ, and rapid tool.

## Figures and Tables

**Table 1:** Origins and visual foliar phenotypes of accessions evaluated for epicuticular waxes by gas chromatography and spectroscopy.

Accession ^z^	‘Cultivar’ and Origin	Phenotype ^y^
B9885	‘White Persian’, Iran	GL
289689	‘Odourless Green Leaf’, Australia	GL
546115	‘White Sweet Spanish Jumbo’, USA	SG
546192	‘Yellow Sweet Spanish’, USA	SG
264320	‘Grano’, Spain	SG
B5351	‘Sweet Spanish Colorado #6′, USDA	SG
DH2107	Cornell University, USA	WX

^z^ Six-digit numbers are plant introductions from the USDA plant germplasm system. Origins of B5351 was described in Damon et al. [25], B9885 in Munaiz and Havey [34], and DH2107 by Hyde et al. [35]. ^y^ Visually scored leaf phenotypes, GL = glossy, SG = semi glossy, and WX = waxy.

**Table 2:** Mean adjusted peak areas with standard errors (SE) for Hentriacontanone-16 (H16), Octacosanol-1 (Oct), and Triacontanol-1 (Tri) measured with gas chromatography mass spectrometry (GCMS) on leaves of plants from seven onion accessions (Table 1) and Tukey’s significance difference. Accessions are ranked from lowest to highest by amounts of H16.

Accession ^z^	Phenotype ^x^	H16	SE		Oct	SE		Tri	SE	
OGL	GL	0.002 ^y^	0.088	a	0.554	0.037	c	0.000	0.025	a
546115	SG	0.532	0.088	b	0.364	0.037	b	0.223	0.025	cd
546192	SG	0.642	0.124	bc	0.454	0.037	bc	0.195	0.025	bcd
B5351	SG	0.648	0.088	b	0.141	0.053	a	0.075	0.036	ab
B9885	GL	0.700	0.088	bc	0.444	0.037	bc	0.140	0.025	bc
264320	SG	1.091	0.088	c	0.586	0.037	c	0.247	0.025	cd
DH2107	WX	2.015	0.088	d	0.496	0.037	bc	0.260	0.025	d

^z^ Origins of accessions listed in Table 1. ^y^ Means followed by the same letter were not significantly different using Tukey’s multiple range test at *p* < 0.05. ^x^ Visually scored leaf phenotypes, GL = glossy, SG = semi glossy, and WX = waxy.

**Table 3:** Percentages of individual waxes on leaves of plants from seven onion accessions measured with GCMS (Table 1). Accessions are ranked from the lowest to highest percentages of H16.

Accession ^z^	Phenotype ^y^	Waxes							
		H16	Oct	Tri	Met	Hex	Octd	Hepc	Hepd
OGL	GL	0.3	70.7	0.0	0.1	19.2	9.6	0.0	0.0
546115	SG	36.8	25.1	15.4	2.8	11.1	0.2	4.8	3.7
546192	SG	40.6	28.7	12.3	3.8	3.2	1.2	6.0	4.1
B5351	SG	52.9	11.5	6.1	5.0	1.1	2.5	12.0	8.9
B9885	GL	44.4	28.2	8.9	2.5	3.8	3.9	3.1	5.2
264320	SG	48.1	25.8	10.9	2.7	4.5	1.1	3.4	3.4
DH2107	WX	65.0	16.0	8.4	1.5	2.4	0.9	2.2	3.6

^z^ Origin of accessions listed in Table 1. ^y^ Waxes are hentriacontanone-16 (H16), octacosanol-1 (Oct), triacontanol-1 (Tri), 2-methyloctacosane (Met), hexacosanol-1 (Hex), 1-ethenyloxy octadecane (Octd), heptacosane (Hepc), and heptadecanol-1 (Hepd) as described by Damon et al. [25]. ^y^ Visually scored leaf phenotypes, GL = glossy, SG = semi glossy, and WX = waxy.

**Table 4:** Spectroscopy validation parameters, R2 values, root-mean-square error (RMSE), range of the interval, and percentage of error within the range for cross calibration and validation models for Hentriacontanone-16 (H16), Octacosanol-1(Oct), and Triacontanol-1 (Tri) on the foliage of seven onion accessions (Table 1).

Wax		Cross Validation				Validation ^y^		
	R^2^	RMSE	%	Range	R^2^	RMSE	%	Range
H16	0.86	0.182	8.25	0–2.20	0.72	0.304	12.62	0–2.41
Oct	0.67	0.108	14.03	0–0.77	0.70	0.102	15.47	0–0.66
Tri	0.48	0.068	16.38	0–0.41	0.41	0.072	20.73	0–0.35

^y^ Validation on an external data set that included the segregating family.

**Table 5:** Spectral features revealed on living tissue with standardized coefficients and VIP for the wavelengths most important for epicuticular waxes Hentriocontanone-16 (H16), Octacosanol-1 (Oct-1), and Triacontanol-1 (Tri1) based on visible-near-infrared to shortwave infrared reflectance (VSWIR, 350–2500 nm) on leaves using a portable spectroradiometer.

Chemical ^y^	Standardized Coefficients ^z^	VIP ^z^
H16 (nm)	*365*, 525, 650, 690, 975, *1233*, *1372*, *1850*, 1900, *2031*, *2082*, *2348,* and *2445*	*365*, 525, 712, 980, 1150,1270, *1372*, *1860*, 1895, 2380, and *2450*
Oct (nm)	*365*, *437*, 550, 680, 750, *1890*, 1950, *2300*, and *2499*	*365*, 550, ***645***, 680, 725, 1000, 1150, *1233*, *1372*, and 1870
Tri (nm)	*365*, 437, 525, *643*, 680, ***730****, 915*, 991, 1150, *1233*, *1372*, *1890*, *2302,* and *2449*	*365*, 680,***730***, 980, 1150, 1328, 1390, *1610*, and *1890*

^y^ Bold numbers indicate unique fingerprints for the corresponding chemical in nanometers (nm). ^z^ Wavelengths in italics aligned with those revealed with each wax chemical standard in Figure 1, Figure 2 and Figure 3, respectively.

**Table 6:** Comparison of chromosome (Chr) and position (Pos) in centiMorgans of the most significant single nucleotide polymorphism (SNP), SNPs flanking the 1.5 logarithm of odds (LOD) confidence interval, percent variation (% Var) explained, LOD threshold (Thresh) values from the permutation analysis, and allelic effects for quantitative trait loci detected by interval mapping of amounts of hentriacontanone-16 (H16), octacosanol-1 (Oct), and triacontanol-1 (Tri) measured by gas chromatography mass spectrometry (GCMS) and predicted by biochemical spectroscopy on foliage of F2 progenies from the cross of glossy B9885 × waxy B8667 onions.

Trait	Chr	Pos	SNP ^z^	1.5 LOD Interval ^z^	% Var	LOD	Thresh	Add ^y^	Dom ^y^
H16 (Spectroscopy)	8	41.1	i19082_1721	i28432_1302-i28633_2705	21.0	4.9	2.9	0.10	0.14
H16 (GCMS)	8	41.1	i19082_1721	i41653_558-i20235_630	45.6	15.8	9.3	0.57	0.33
Oct (Spectroscopy)	8	41.1	i19082_1721	i29044_2564-i28633_2705	21.0	3.8	3.5	0.36	0.31
Oct (GCMS)	8	46.2	i20235_630	i19082_1721–i28633_2705	16.9	3.7	3.5	0.36	0.31
Tri (Spectroscopy)	8	41.1	i19082_1721	i41653_558-i20235_630	36.4	9.1	3.6	0.29	0.28
Tri (GCMS)	8	46.2	i20235_630	i19082_1721–i28633_2705	23.3	5.5	3.6	0.30	0.19

^z^ i = names and map positions of SNPs were reported by Munaiz and Havey [34]. ^y^ Significant additive (Add) and dominance (Dom) effects of allele from the waxy parent (B8667) that increased the amounts.

## Supplementary Materials

The following are available online, Figure S1: Density plots comparing mean amounts ± standard errors of fatty alcohols (Octacosanol-1(Oct, left) and Triacontanol-1 (Tri, center)) and ketone Hentriacontanone-16 (H16, right) from GCMS and spectroscopy. Y-axes on the left are for plots of Oct and Tri, and the *y*-axis on the right for H16. Horizontal axis shows amounts of wax component. Vertical dashed lines show the median for GCMS (blue) and Spectroscopy (yellow). Figure S2. Scatter plot of the best regression for observed and predicted H16 values (top) and plot of residuals and observed values (bottom).
