# Supplementary material for: Reflectance Spectroscopy for Non-Destructive Measurement and Genetic Analysis of Amounts and Types of Epicuticular Waxes on Onion Leaves
Source: Molecules. 2020 Jul 29;25(15):3454. doi: 10.3390/molecules25153454 (PMC7436246; doi:10.3390/molecules25153454)
Supplement: Supplementary file 1 [file molecules-25-03454-s001.pdf]

Article

Reflectance Spectroscopy for Non-Destructive Measurement and Genetic Analysis of Amounts and Types of Epicuticular Waxes on Onion Leaves

Eduardo D. Munaiz [1], Philip A. Townsend [2] and Michael J. Havey [3]*

[1] Department of Horticulture, University of Wisconsin, 1575 Linden Drive, Madison, WI 53706 USA; dominguezmun@wisc.edu
[2] Department of Forestry and Wildlife Ecology, University of Wisconsin, 1575 Linden Drive, Madison, WI 53706 USA; ptownsend@wisc.edu
[3] USDA-ARS and Department of Horticulture, University of Wisconsin, 1575 Linden Drive, Madison, WI 53706 USA; michael.havey@usda.gov
* Correspondence: michael.havey@usda.gov;

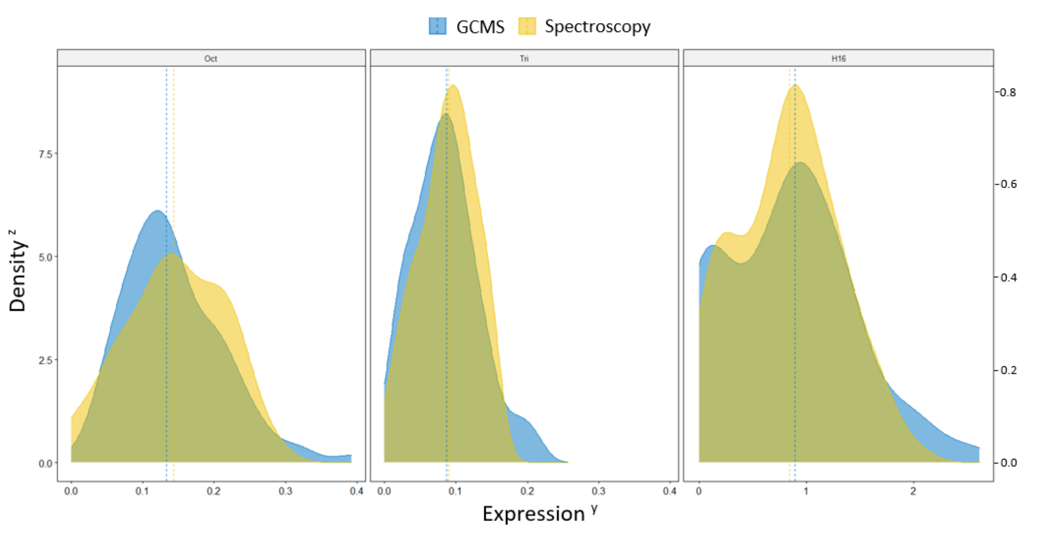

Methodology	Oct	Tri	H16
GCMS	0.143 ± 0.063	0.088 ± 0.004	0.837 ± 0.063
Spectroscopy	0.145 ± 0.007	0.085 ± 0.005	0.826 ± 0.049

Supplementary Figure S1. Density plots comparing mean amounts ± standard errors of fatty alcohols [Octacosanol-1(Oct, left) and Triacontanol-1 (Tri, center)] and ketone Hentriacontanone-16 (H16, right) from GCMS and spectroscopy. Y-axes on the left are for plots of Oct and Tri, and the y-axis on the right for H16. Horizontal axis shows amounts of wax component. Vertical dashed lines show the median for GCMS (blue) and Spectroscopy (yellow).

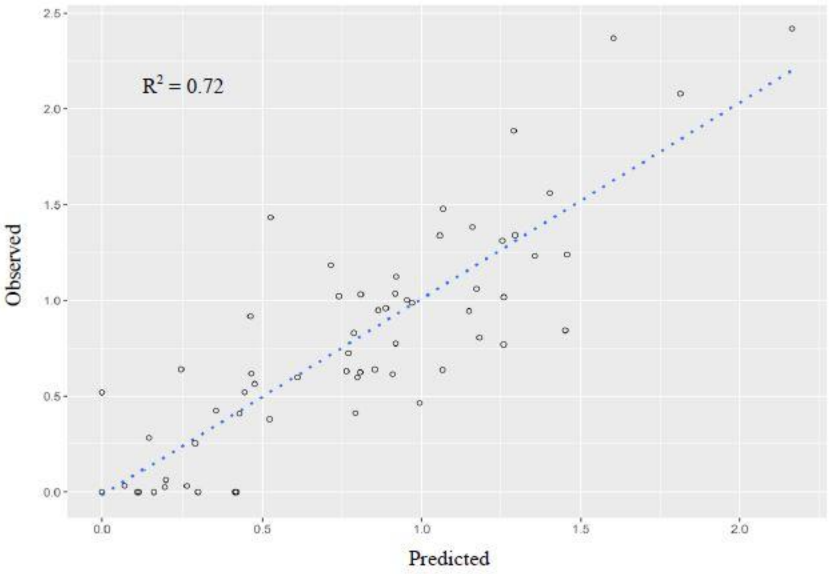

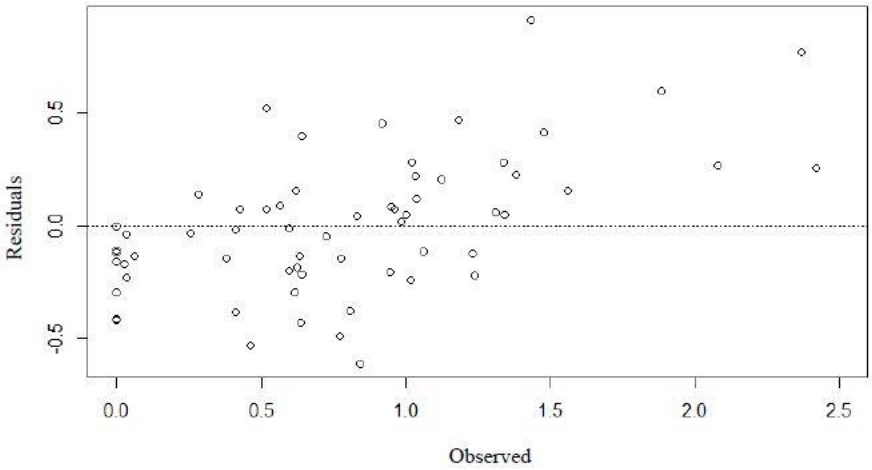

Supplementary Figure S2. Scatter plot of the best regression for observed and predicted H16 values (top) and plot of residuals and observed values (bottom). .
